# Primary pulmonary alveolar soft part sarcoma with ASPSCR1–TFE3 gene fusion: Case report and literature review

**DOI:** 10.1097/MD.0000000000040249

**Published:** 2024-11-01

**Authors:** Xijian Hu, Jing Chai, Bin Zhang, Chengguang Hu

**Affiliations:** a Graduate School, Shanxi Medical University, Taiyuan, China; b Department of Thoracic Surgery, Shanxi Cancer Hospital, Taiyuan, China.

**Keywords:** alveolar soft part sarcoma, ASPSCR1–TFE3 gene fusion, case report, pulmonary

## Abstract

**Rationale:**

Primary pulmonary alveolar soft part sarcoma (ASPS) is an extremely rare disease characterized by a specific genetic abnormality – the ASPSCR1-TFE3 gene fusion.

**Patient concerns:**

This study presented a 27-year-old male patient who experienced persistent chest tightness for over 6 months.

**Diagnoses:**

The computed tomography (CT) scan and enhanced CT scan revealed a mass in the medial segment of the right middle lobe of his lung. The patients then underwent further diagnosis. Pathological examination showed the tumor to be consisting of polygonal cells with abundant eosinophilic or transparent cytoplasm arranged in nests. Next-generation sequencing reported ASPSCR1-TFE3 gene fusion, confirming the final diagnosis of primary pulmonary ASPS. Regular follow-ups of 12 months showed no signs of tumor recurrence.

**Interventions:**

The patients underwent the medial segment resection of the right middle lobe for treatment.

**Outcomes:**

A CT examination 3 months after the operation showed that the patient had improved. The last review showed no recurrence or metastasis.

**Lessons:**

This case report highlights the importance of detailed diagnosis, prompt treatment, and close monitoring of patients with ASPS.

## 1. Introduction

Alveolar soft part sarcoma (ASPS) is a rare tumor of uncertain histogenesis, accounting for only 0.5% to 1% of all soft tissue tumors.^[1]^ The overall 2-year survival rate is 82%, and 5-year survival rate is 56%.^[2]^ ASPS is characterized by der(17)t(X;17)(p11;q25) resulting in ASPSCR1–TFE3 fusion.^[3]^ Demonstration of this particular gene rearrangement is the most desirable diagnostic criterion of ASPS. Conventional chemotherapy and radiation therapy are not beneficial in most previous studies.^[4]^ Surgical excision is the main treatment method for ASPS, and has been reported to improve the prognosis of both patients with localized disease and distant metastases.^[2]^ Primary pulmonary ASPS is extremely rare. Literature to date reported only 6 instances of primary pulmonary ASPS.^[5–10]^ Due to financial and technical considerations, only 2 of the 6 cases confirmed the presence of ASPSCR1–TFE3 fusion.^[6,7]^ This study reported a 27-year-old male case of primary pulmonary ASPS with confirmed ASPSCR1–TFE3 fusion and reviewed the currently published literature.

## 2. Case presentation

**Figure 1.**
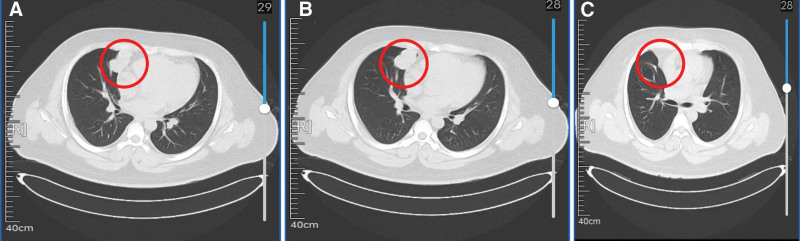
CT scan images during treatment. (A) CT scan upon admission (November 9, 2022); (B) The enhanced CT scan before the surgery (November 9, 2022); (C) CT reexamination 3 months after the surgery (March 23, 2023). CT = computed tomography.

On November 10, 2022, a 27-year-old male patient visited the Department of Thoracic Surgery, Shanxi Cancer Hospital due to persistent chest tightness for more than 6 months. No familial history of similar conditions was noted, and physical examination revealed no palpable superficial lymph nodes. The patient showed an elevated tumor marker tissue polypeptide-specific antigen of 233.71 U/L (normal reference range: <150 U/L). A computed tomography (CT) scan revealed a high-density shadow in the medial segment of the right middle lobe of his lung (Fig. 1A). The enhanced CT scan revealed a mass with a distinct border, displaying lobulated and smooth edges, measuring approximately 4.7 × 2.7 cm, and reported a suspicion of a sclerosing pulmonary cell tumor (Fig. 1B). Preoperative cranial magnetic resonance imaging, abdominal and cervical ultrasound, whole-body bone scintigraphy, and other examinations showed no abnormalities, indicating the lung to be the primary site of the tumor, and no detectable distant metastasis.

**Figure 2.**
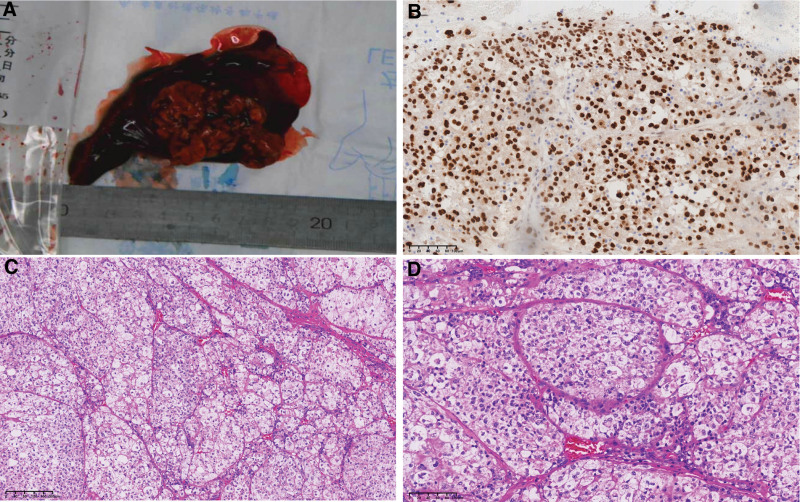
Histopathological findings from the biopsy specimen. (A) Macroscopic view of the surgical specimen. The resected mass from the right lung measured 4.5 × 2.5 cm and exhibited a grayish-yellow hue with a soft texture and an ill-defined boundary; (B) Positive expression of TFE3 in tumor cell nuclei; (C) Moderately enlarged tumor tissue organized in nests, with narrow sinus lumens separating the tumor cell nests (HE, 100×); (D) Nested distribution of the tumor tissue (HE, 200×).

**Figure 3.**
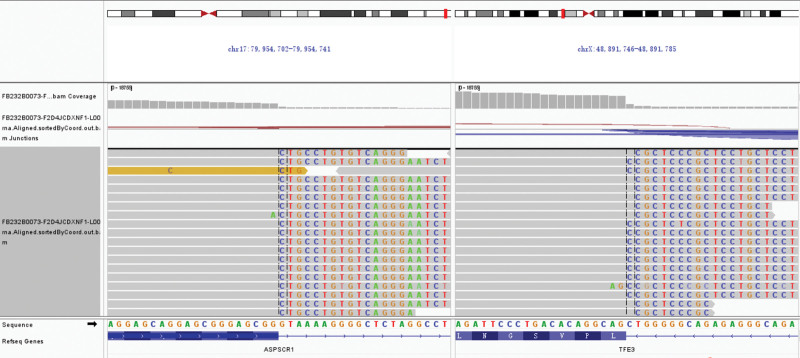
Whole genome sequencing (RNA) of ASPSCR1 on chr17: 79,954,702–79,954,741 and TFE3 on chrX: 48,891,746–48,891,785.

The patients then underwent the medial segment resection of the right middle lobe for treatment and further diagnosis. Intraoperative frozen section diagnosis revealed epithelioid cells with abundant cytoplasm arranged in nests, and no definitive diagnosis was made at the time. The paraffin section was then prepared and observed. Microscopically, the tumor exhibited a solid growth pattern, consisting of polygonal cells with abundant eosinophilic or transparent cytoplasm arranged in nests. These tumor cell nests were separated by narrow sinus lumens. Certain regions displayed atypical nuclei, distinct nucleoli, and an absence of necrosis (Fig. 2). Immunohistochemistry showed TFE3(+), Ki67(about 8%+), S-100(focal+), CD68(portion+), TTF-1(‐), AE1/AE3(‐), HMB45(‐), P40(‐), SMA(‐), Desmin(‐), CA-IX(+), IN-1(+), and SMARCA4(+), suggesting that the tumor could be associated with TFE3 gene translocation. Next-generation sequencing (NGS) sequencing reported ASPSCR1–TFE3 gene fusion, ASPSCR1: exon7–TFE3: exon6 (abundance 14.20%) (Fig. 3). These results confirmed the diagnosis of primary pulmonary ASPS, with ASPSCR1–TFE3 gene fusion.

The CT scan 3 months after the surgery suggested minor inflammatory changes in the right lung and punctate calcification in the lower lobe of the right lung (Fig. 1C). The patient was followed up regularly after discharge, once every 3 months, and the last review was in December 2023, with no recurrence or metastasis.

## 3. Discussion

This study reported a male case of primary pulmonary ASPS with the ASPSCR1–TFE3 gene fusion.

Primary pulmonary ASPS affects the lungs specifically and accounts for <1% of all lung tumors.^[11,12]^ A literature review of primary pulmonary ASPS was conducted, only 6 cases had been reported so far,^[5–7,9,10,13]^ and only 2 of which had confirmed the presence of ASPSCR1–TFE3 fusion.^[6,7]^ To the best of our knowledge, this was the third case of primary pulmonary ASPS with confirmed ASPSCR1–TFE3 fusion, and the clinical characteristics of the 3 cases were presented in Table 1. There were more males than females (2:1), with ages ranging from 27 to 48 years. The patient in one previous case showed chest pain, while the other one was asymptomatic. Pathological examination found polygonal tumor cells with abundant eosinophilic or transparent cytoplasm arranged in nests in the 2 reported cases, which was similar to ours. In all 3 cases, immunohistochemistry revealed a strong positive TFE3 stain, and NGS identified the ASPSCR1::TFE3 fusion gene. Each case underwent complete tumor removal surgery, and follow-ups showed no evidence of local recurrence or distant metastasis.

**Table 1:** Summary of 3 primary pulmonary ASPS with confirmed ASPSCR1–TFE3 fusion.

First author, year	Sex	Age	Symptoms	CT	Pathological examination	IHC	NGS	Distant metastasis	Treatment	Follow-up
Jiangying Zhao (2023)^[6]^	M	30	Chest pain and dyspnea	A mass in the left lung	Large, round, or polygonal tumor cells, with clear boundaries, abundant cytoplasm, and eosinophilic granules	TFE3(+)	ASPSCR1::TFE3 fusion gene	None	Surgery	No recurrence or metastasis was detected after 6 months
Ming Zhao (2015)^[7]^	F	48	None	A mass at the hilum of the left lung	Large, polygonal to round tumor cells, and discohesive with voluminous eosinophilic, to pale, to clear cytoplasm	TFE3(+)	ASPSCR1::TFE3 fusion gene	None	Surgery	No recurrence or metastasis was detected after 12 months
Present report	M	27	Chest tightness	A mass in the middle lobe of the right lung	Polygonal cells with abundant eosinophilic or transparent cytoplasm arranged in nests	TFE3(+)	ASPSCR1::TFE3 fusion gene	None	Surgery	No recurrence or metastasis was detected after 12 months

ASPS = alveolar soft part sarcoma, CT = computed tomography, F = female, IHC = immunohistochemistry, M = male, NGS = next-generation sequencing.

Symptoms of primary pulmonary ASPS can include coughing, chest pain, shortness of breath, and fatigue, but these are nonspecific. Diagnosis typically involves imaging studies such as CT scans and magnetic resonance imaging, but interpreting the results can be challenging due to the rarity of the disease. Treatment options for primary pulmonary ASPS are limited and primarily involve surgical resection for localized disease.^[13]^ The effectiveness of chemotherapy and radiation therapy remains to be determined due to a lack of clinical trials. Unfortunately, the prognosis for primary pulmonary ASPS is generally poor, with a high likelihood of recurrence and metastasis.

A defining characteristic of ASPS is the presence of a specific genetic abnormality—the ASPSCR1–TFE3 gene fusion—which results from a translocation between chromosomes X and 17.^[8,11]^ This fusion gene promotes the growth and survival of cancer cells, leading to the development of ASPS. The ASPSCR1–TFE3 fusion protein acts as a transcription factor, regulating gene expression in cell proliferation, differentiation, and survival. Several studies have explored the molecular mechanisms behind the formation and function of this fusion protein,^[8,14–16]^ providing evidence that the ASPSCR1–TFE3 fusion protein plays a crucial role in the development and progression of ASPS by promoting angiogenesis, regulating cell cycle progression, and modulating the transcription of genes involved in lipid metabolism. Tanaka et al discussed how the ASPSCR1::TFE3 fusion protein orchestrated the angiogenic program of ASPS by modulating the activity of super-enhancers associated with genes involved in angiogenesis.^[14]^ Sun et al described the genomic and transcriptomic features of TFE3-translocation renal cell carcinoma, including the association between ASPSCR1–TFE3 fusion and aggressive features and poor outcomes.^[15]^ Another research reported on detecting the ASPSCR1–TFE3 gene fusion in paraffin-embedded alveolar soft part sarcomas using fluorescence in situ hybridization.^[8]^ The role of the ASPL–TFE3 oncoprotein in regulating cell cycle progression and transcription of genes involved in lipid metabolism in ASPS has also been identified.^[16]^

NGS sequencing of RNA Capseq panel and fusion detection contribute to diagnosing ASPS,^[17]^ allowing for the rapid and comprehensive analysis of the genetic alterations. The RNA Capseq panel is a targeted sequencing approach that focuses on the coding regions of genes involved in cancer. This panel includes genes frequently mutated or rearranged in various types of cancer, including ASPS. Researchers can identify the genetic alterations driving cancer development by sequencing the RNA from tumor samples using the Capseq panel. By detecting the ASPSCR1–TFE3 fusion gene using NGS sequencing of RNA Capseq panel, clinicians can confirm the diagnosis of ASPS and develop more targeted and effective treatments for patients. Several studies have demonstrated the utility of this technique in diagnosing and characterizing ASPS. One study developed and validated a custom-designed RNA sequencing panel including 67 genes to identify STS gene fusions containing more than 70 subtypes.^[18]^ NGS sequencing with Capseq panel is a crucial tool in diagnosing this rare and challenging tumor.

## 4. Conclusion

In conclusion, ASPS with ASPSCR1–TFE3 gene fusion is a rare cancer primarily affecting young patients. Treatment options for this type of cancer are limited, and surgical resection remains the gold standard. This case report highlights the importance of close surveillance in patients with ASPS, even in cases where there is no evidence of tumor recurrence.

## Author contributions

**Data curation:** Xijian Hu, Jing Chai, Bin Zhang.

**Formal analysis:** Xijian Hu, Jing Chai, Bin Zhang.

**Writing – original draft:** Xijian Hu, Chengguang Hu.

**Writing – review & editing:** Chengguang Hu.
